# Investigation of the effectiveness of the “Girls on the Go!” program for building self-esteem in young women: trial protocol

**DOI:** 10.1186/2193-1801-2-683

**Published:** 2013-12-19

**Authors:** Loredana Tirlea, Helen Truby, Terry P Haines

**Affiliations:** Department of Nutrition and Dietetics, Southern Clinical School, Faculty of Medicine, Nursing and Health Sciences, Monash University, Level 1, 264 Ferntree Gully Road, Notting Hill, 3168 Victoria Melbourne, Australia; Monash Health, (formerly Southern Health) Greater Dandenong Community Health Services, Springvale, Melbourne, Australia; Department of Physiotherapy, Southern Physiotherapy Clinical School, Monash University, Frankston, Melbourne, Australia; Monash Health, (formerly Southern Health) Allied Health Research Unit, Kingston, Melbourne, Australia

**Keywords:** Self-esteem, Body image, Disordered eating, Eating disorders, Community health intervention, Health promotion, Group therapy, Girls, Young women, Adolescents

## Abstract

**Background:**

Body Image is a major factor affecting health in a range of age groups, but has particular significance for adolescents. The aim of this research is to evaluate the efficacy of the “Girls on the Go!” program delivered outside of the school environment by health professionals to girls at risk of developing poor self-esteem on the outcomes of self-esteem, impairment induced by eating disorders, body satisfaction, self-efficacy, and dieting behaviour.

**Method:**

A stepped wedge, cluster randomised controlled trial that was conducted in two phases on the basis of student population (Study 1 = secondary school age participants; Study 2 = primary school age participants). The waiting list for the “Girls on the Go!” program was used to generate the control periods. A total of 12 schools that requested the program were separated into study 1 or 2 on the basis of student population (Study 1 = secondary, Study 2 = primary). Schools were matched on the basis of number of students and were allocated to receiving the intervention immediately or having a waiting list period. Study 1 had one waiting list period of one school term, creating two steps in the stepped-wedge design (i.e. 3 schools were provided with “Girls on the Go!” each term over 2 terms). Study 2 had two waiting list periods of one and two school terms, creating three steps in the stepped-wedge design (i.e. 2 schools were provided with “Girls on the Go!” each term over 3 terms). Primary outcome measures were self-esteem and impairment inducted by eating disorders.

**Discussion:**

There is a lack of preventative interventions currently available that address low self-esteem, low self-efficacy and body dissatisfaction in young women. This project will be the first group-based, professional-led, targeted program conducted outside the school environment amongst school age young women to be evaluated via a randomised control trial. These findings will indicate if the “Girls on the Go!” program may be successfully used and applied in a culturally diverse environment and with young women of all shapes and sizes.

**Trial registration:**

(ACTRN12610000513011)

**Electronic supplementary material:**

The online version of this article (doi:10.1186/2193-1801-2-683) contains supplementary material, which is available to authorized users.

## Background

Body image is the “mental picture” we form of our physical body and encompasses how we feel and think about our physical appearance (Bak-Sosnowska, 2008). Poor body image is common amongst both young men and women, but particularly so amongst young women (Paxton, 2000). Self-esteem is the overall evaluation of our worth, including how we like, respect, and accept ourselves (Mann et al., 2004). Another related self-concept, self-efficacy refers to the confidence we place in ourselves that we possess the ability and skills necessary to exercise control over our daily lives (Bandura, 1986). Due to society’s strong emphasis on appearance, many young people are confused about what self-esteem is and many tend to measure and value their self-worth by their shape, weight, appearance and material assets (O’Dea, 2004; Mann et al., 2004).

Positive relationships have been identified between poor body image and low self-esteem and health problems such as eating disorders (Chisuwa and O’Dea, 2010; Ata, 2003; Breines et al., 2008; O’Dea and Abraham, 2000; Paxton et al., 2006; Stice, 2002; Graber et al., 1994), depression and other mental health issues (Ambresin et al., 2012), obesity (Bak-Sosnowska, 2008), and being physically inactive (Huang et al., 2007). Also, poor body image has been associated with unhealthy weight loss practices such as engaging in extreme dieting behaviours like purging, and also binge eating (Heinicke et al., 2007; Paxton, 2000; Paxton, 1993). Links have been reported between weight loss practices and further weight gain (Stice et al., 1999) and this vicious cycle is believed to contribute to the child obesity epidemic (O’Dea, 2004). Some recent research now supports the notion that weight loss is only achieved short-term and food restriction leads to more weight gain, poorer self-esteem, eating disorders and overall poor health outcomes (Bacon and Aphramor, 2011; Bacon et al., 2005; Stice et al., 1999). Thus, the relationships between self-esteem, body image and dietary behaviours are complex and multi-faced. Indeed previous authors have described the effects of poor body image on emotional well-being and self-esteem as a ‘ripple effect’ that impacts on health (Healey, 2003, p.5).

Health education programs which enhance protective self-concepts such as self-esteem, self-efficacy are needed, in particular for young women. It is possible that incorporating health promoting activities within intervention programs designed to build self-esteem can be critical in addressing these inter-related problems as high self-esteem has been found to be a protective factor against body dissatisfaction and disordered eating (McVey et al., 2003, 2002; Wade et al., 2003; Button et al., 1996; O’Dea and Abraham, 2000; Smolak, 2009; O’Dea, 2004; Albee, 1996; Bayer, 1984; Thompson and Smolak, 2001; Shisslak et al., 2001). An approach focussed on addressing low self-esteem has been used by previous programs that aimed to improve body image and reduce eating problems amongst young children and adolescents (Steese et al., 2006; O’Dea, 2000, 2004; Steiner-Adair et al., 2002; Neumark-Sztainer et al., 2000; Wade et al., 2003; McVey et al., 2004; O’Dea and Abraham, 2000; McVey, 2002; Richardson et al., 2009; Phelps et al., 2000; Kater et al., 2002; Smolak and Levine, 2001). These programs have generated promising though mixed results in improving a range of health amongst young women. Programs that promote improved self-esteem, self-efficacy and body image, reduced the thin idealisation while mitigating risk factors for eating disorders achieved short term effects (O’Dea, 2004; Steese et al., 2006; O’Dea and Abraham, 2000), though some similar programs did not demonstrate sustained benefits in the long term (McVey et al., 2004; Stewart et al., 2001), and others did not demonstrate any desired benefits (Wade et al., 2003; Paxton, 1993).

Programs designed to improve self-esteem, self-efficacy and body satisfaction, among young women can be categorised in many ways. This includes categorising according to the target population (e.g. Universal versus targeted program delivery), the setting of delivery (e.g. within or outside the school/classroom setting vs. community setting), whether the program is a group or individual (counselling) program, and the facilitation method of the program (health professional, peer or teacher led) (Stice and Shaw, 2004; Paxton, 2002, 2011; Levine and Smolak, 2009). There are potential advantages and disadvantages of each of these delivery methods. Delivering eating disorder prevention programs within the school environment allows large numbers of students to be readily provided with the program. However, previous research has generated mixed results as to whether programs delivered by trained peer leaders and teachers in the school environment produce positive outcomes (Becker et al., 2006; Marchand et al., 2011; Stice et al., 2009) as compared to negative outcomes (Carter et al., 1997; O’Dea, 2002, 2000b). One delivery issue that may be an important factor here is whether programs have been delivered to entire school populations or to selected sub-groups of students who are identified as being at higher-risk for developing poor self-esteem and other related negative outcomes. Delivery to only those at high risk may be a more cost-effective approach as resources are not being wasted on those who do not need them, while delivery of programs outside of the school environment permit privacy and allow students to be taken outside the setting which may be contributing to these problems. Further to this, programs led by peers or teachers may not facilitate open and honest discourse due to pre-existing relationships with the participants. Several meta-analyses (Stice and Shaw, 2004; Stice et al., 2007; Cororve Fingeret et al., 2006) have reported that for those at ‘high risk’ end of the spectrum interventions provided by trained professionals rather than teachers were more effective in obtaining long lasting and positive outcomes.

Much of the previous research that has been conducted has not targeted girls at higher risk of developing poor self-esteem and related health outcomes amongst those under the age of 15, and it is less certain whether programs such as those previously investigated are beneficial when delivered to these younger women. This research aims to determine the efficacy of a community-based, health professional-run program to improve self-esteem and related health outcomes amongst girls who have been identified as being at risk of developing eating disorders, body dissatisfaction, or extreme dieting behaviour. The hypothesis to be tested is that participants exposed to this program will experience improvement in self-esteem, mental health self-efficacy, physical health self-efficacy, body satisfaction, dieting behaviour and they will experience less impairment induced by eating disorders relative to those exposed to the waiting-list control condition. Age is a salient factor for prevention interventions designed to minimise future ‘risk’, for example early intervention and skill training are recommended for resilience building (Gillham and Reivich, 2010) (Zolkoski and Bullock, 2012). Therefore, this larger project incorporated two separate studies. Study one investigates the efficacy of the program with high school age participants. Study two investigates the efficacy of the program with primary school age participants.

### Design

This study comprised a stepped wedge, cluster randomised controlled trial that was conducted in two phases on the basis of student population (Study 1 = secondary school age participants; Study 2 = primary school age participants). Stepped wedge designs are similar to cross-over trials, however only involve uni-directional cross-over resulting in all participants receiving the intervention by the conclusion of the trial. The waiting list for the “Girls on the Go!” program was used to generate the control period. The intervention program was designed to be delivered to a group of participants from the same school at the same time, thus randomising students in clusters (school) was seen as an appropriate unit of randomisation.

**Figure 1:**
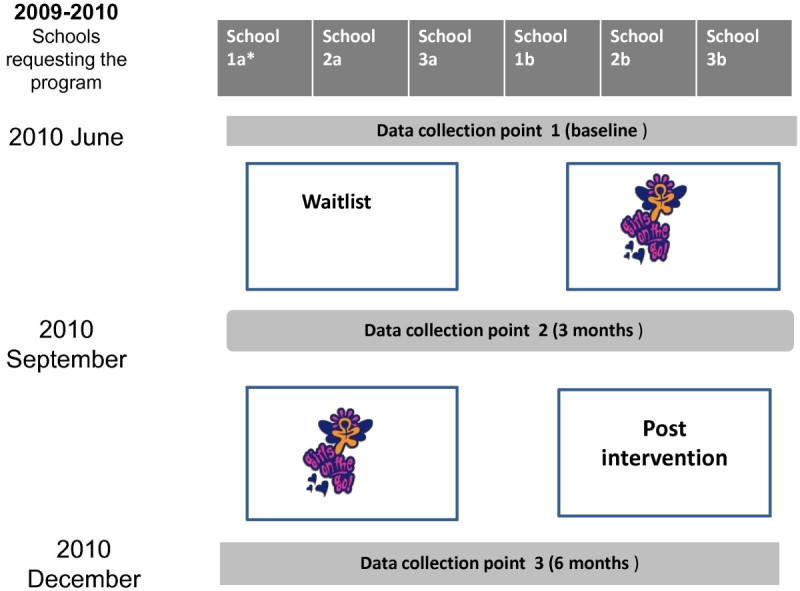
**Stepped-wedge design for Study 1.** Note: *School 1a and 1b, 2a and 2b, and 3a and 3b were paired and matched based on size and randomly allocated to either waitlist or intervention.

**Figure 2:**
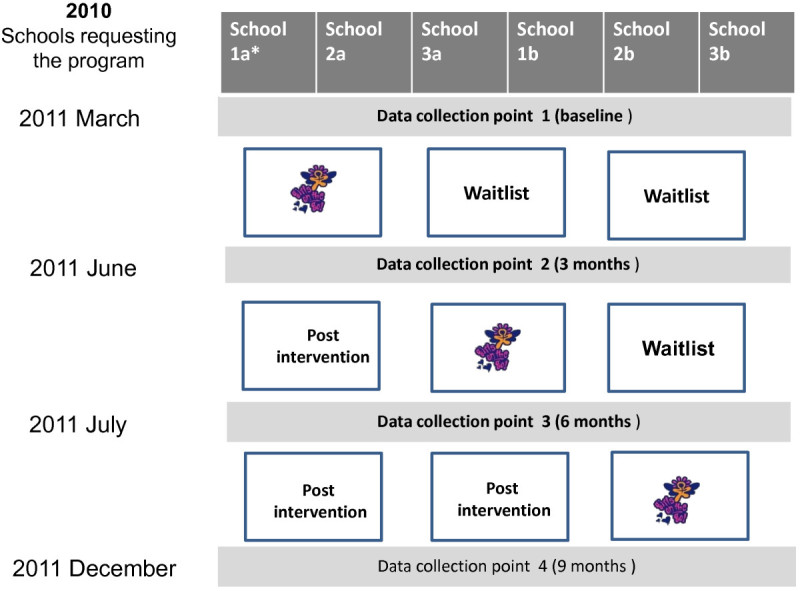
**Stepped-wedge design for Study 2.** Note: *School 1a and 1b, 2a and 2b, 3a and 3b were matched based on size paired and randomly allocated to either waitlist or intervention.

A total of 12 schools that requested the program were separated into Study 1 or 2. Within each study, schools were matched on the basis of number of students (into triplets for Study 1 and pairs for Study 2) and then randomly allocated (computer generated sequence developed by investigator blinded to school identity) to receiving the intervention immediately or having a waiting list period. Study 1 had one waiting list period of one school term, creating two steps in the stepped-wedge design (i.e. 3 schools were provided with “Girls on the Go!” each term over 2 terms–see Figure 1). Study 2 had two waiting list periods of one and two school terms, creating three steps in the stepped-wedge design (i.e. 2 schools were provided with “Girls on the Go!” each term over 3 terms–see Figure 2).

### Participants and setting

Participants in this study were either high school or primary school students who were referred to the Greater Dandenong Community Health Centre to receive the intervention program. The intervention was a program being run prior to the conduct of this trial and schools in the City of Greater Dandenong municipality had been routinely referring students to this program for the previous 8 years. Each school group consisted of between 8–12 participants.

### Inclusion criteria

Eligibility criteria of young women to be referred to the program and also to be included in the study were: poor or negative body image, low self-esteem, inactivity in sports and exercise, poor diet, or being overweight or underweight. Participants were between 10 and 17 years of age. These students needed to be identified and referred by their school welfare officer or teacher.

### Local contextual considerations

The City of Greater Dandenong is the most culturally diverse locality in Victoria, Australia, with residents from 156 different birthplaces, over half (56%) of its population born overseas, and 51% from nations where English is not the main spoken language. An influx of approximately 2300 newly arrived settlers per year since 2006 has sustained this diversity. The City of Greater Dandenong has also been described as one of the lowest socio-economic indexes for (SIEFA) Melbourne (SIEFA indices allow ranking of regions/areas by providing a method of identifying the level of social and economic well-being in each region) (ABS, 2011).

### Intervention

The community-based, health professional led program that formed the intervention for this research is called the “Girls on the Go!” program.

## “Girls on the Go!” program development

A Youth Community Health Team in Melbourne, Australia, developed the “*Girls on the Go!*” in response to an identified need and demand by local schools and communities for a program designed to address body dissatisfaction and promote healthier lifestyles. The program developers were unable to model elements of this program on what was known to be successful in 2001 due to a paucity of evidence from experimental studies investigating the efficacy of programs to improve self-esteem in young women. Indeed the concept of using programs to enhance self-esteem in order to minimise disordered eating was only proposed in the late 1990s (O’Dea and Abraham, 2000).

Following a successful pilot program, a forum was held which included local schools and youth agencies which resulted in further promotion of the existence of the program through word of mouth. It was intended “*Girls on the Go!*” target young women who have been identified to be at high risk of a variety of eating and body image difficulties, poor self-esteem, low self-efficacy, negative self-image and physical inactivity at an early stage so they do not develop a serious mental illness e.g. anorexia/bulimia nervosa, or physical illness e.g. diabetes, heart conditions, later in life.

The program was initially developed by a multidisciplinary team of health practitioners including a Social Worker, Sport and Recreational Worker, and a Community Health Nurse. These workers had specific skills pertaining to their discipline and their area of expertise was used to contribute to separate components of the program. For example, the Social Worker contributed counselling specific skills to the program in general but also contributed more specific information to the session “A healthy mind” and “Body Image and Self Esteem” as well as providing the initial link with the young person as someone they know and could contact later should they require additional one on one support. The Sports and Recreational worker provided their expertise into designing the physical exercise components of the program as well as linking into their networks and contacts from the Sports and Recreational Industry. Previously these workers had been independently running programs to address health and wellbeing issues such as self-esteem, self-efficacy, body image, mental health, stress and relaxation, healthy lifestyles, healthy eating and being physically active. However, they sought to integrate a combined program of activities that could be conducted in a group context.

### Theoretical approach underpinning the development of “Girls on the Go!”

The initial stages of the “*Girls on the Go!*” program development were driven more from practical experience. However the founders of the program came from various disciplines (i.e. sport and recreational work, social work, youth work, and nursing) that can be placed within the allied health spectrum and the program was set up within a primary health setting. Hence similarities existed in their approach to working with young people. The program was developed therefore, with an overarching theme of engaging young women in a holistic manner within an early intervention bio/psycho/social health model (Engel, 1977).

The bio-psycho-social model of health is a theoretical model used for considering individual and population health and wellbeing. It operates under the presumption that improved health and well-being may be achieved by targeting the determinants of health including social and environmental as well as the biological and medical factors. Working within the social model of health is advantageous because it permits not only individuals but entire communities to define health according to their understanding of it (Keleher and Marshall, 2002). Therefore, it empowers them to identify important factors that influence their own health depending on their specific context. The social model of health proposes that physical, mental and social health is interlinked and play an equal role in wellbeing. This is especially relevant when considering the multiple influences and risk factors of disordered eating. The individuals’ self-concept is greatly impacted by each of these determinants and the social model of health gives a holistic and well balanced framework to develop an effective program.

### Program description–content

“*Girls of the Go!*” provides a structured, community-based, half-day, 10 week health promotion program for a group of up to 10 young women that uses self-esteem enhancement, to improve body satisfaction, self-efficacy, and promote healthy lifestyles including healthy eating and reduce dieting behaviour and promote positive attitudes towards sport and exercise. The intervention encourages information sharing and providing support to one another which leads to strong relationships forming amongst members of the group. Participants share their similar experiences and this draws them closer to one another.

Positive mental and physical health is promoted via education about healthy lifestyle choices, building self-esteem and self-efficacy, improving body dissatisfaction via media literacy, participating in fun activities and developing community linkages for participants. Youth Workers deliver the program with other health professional such as dieticians and physiotherapists supporting the program as needed.

Active participant involvement in planning program activities is incorporated to empower participants to make decisions about their own health to make the continuation of positive practices more likely at the completion of the group. Participants engage in activities and discussion designed to address a particular topic. Elements of the program and duration may be modified according to the needs of the target group, skills, experience, equipment and facility availability. The program was initially developed for a community health setting but has since been expanded to youth agencies, neighbourhood houses, local hospital as well as main stream and non-mainstream schools. To ensure fidelity of the program delivered, all facilitators must undergo formal training and also observe one full program before being able to lead a program on their own. The program has always been delivered from a community health centre setting and outside the school classroom environment.

The “Girls on the Go!” program is delivered over a ten week period with a total of 26 contact hours over the life of the program consisting of interactive discussion and activities (See Table 1).

**Table 1:** **“Girls on the Go!” curriculum outline**

Session	Duration	Theme	Aim and content
Session 1	1 hour	Welcome to “Girls on the Go!”	This session outlines the aims of the program and educates the participants on what each of the subsequent sessions would focus on.
Session 2	3 hours	Let’s get to know each other	The participants are given a tour of the Health Centre and are introduced to all members of the facilitator team. The session employs team building games and activities that encourage the participants to learn more about one another and their facilitators, and to become comfortable in the group environment. Goal setting regarding exercise is performed during this session to create a goal for the participants to work towards during the program. Realistic goals are emphasised to encourage feelings of competence and self-determination.
Session 3	3 hours	Body Image and Self-Esteem	To help participants understand the concept of Body Image and related issues. The session involves discussions and a number of activities designed to emphasize how a range of factors can affect Body Image.
Session 4	3 hours	Personal Safety and Assertiveness	The session incorporates the themes of safety and assertiveness with a guest presenter from local police covering topics such as personal safety in public spaces and safe partying for teenagers; presentation on stranger danger for younger age participants and public transport safety; this is followed by another presenter teaching the participants basic self-defence techniques encompassed in Tae Kwon Do.
Session 6	3 hours	A Healthy Mind	To emphasise the importance of mental health, especially during teenage years, and to highlight some methods of managing mental health. Guest speakers inform the participants about others experiences and ways in which to manage stress and adverse thoughts. It involves group participation in a discussion followed by a Yoga Class.
Session 7	3 hours	Physical activity	To help participants understand the importance of physical activity and also to introduce them to a range of activities they may not have thought of before. Allows participants to nominate a form of physical activity which they believe would be of benefit to them. This specific part of the program is to build upon an empowerment model, encouraging the participants to hold responsibility for their own participation in exercise by allowing them to actively plan and implement the session.
Session 8	3 hours	Trust and Confidence	Aim for participants to extend the trust and support they have for each other. Participants take part in an indoor rock climbing activity. Rock climbing is an activity that endorses team work and initiates the formation of new skills. Peer support is promoted throughout the session as the other participants are in control of the individual’s guide ropes.
Session 9	6 hours	Celebration	To reflect on what the participants have learned throughout the program and to celebrate these achievements. The session delivers closure to the program and educates the participants on the ways they can remain healthy and active in the community.
Session 10	1 hour	Connections	The aim of this session is to educate the participants on how to further apply what they had learnt during the program in their school and community, such as organising awareness workshops regarding self-esteem and body image that the participants run through their school.

### Pilot of the “Girls on the Go!” intervention

A retrospective program review of the data collected from participants between 2001 and 2009 was conducted. Ethical approval was received by the Southern Health’s Research Directorate (Research Project number: 09252Q, HREC09252). Questions examining self-esteem, self-efficacy (responses captured using 5 point visual analogue scale), and satisfaction with body image (responses captured using 4 point visual analogue scale) were compared between pre and post program assessments using ordered logit regression analysis with clustering by participant to account for dependency between pre and post assessments within participant.

Ordered logit regression calculates the increase or decrease in odds of moving up one level in the ordinal dependent variable associated with a one unit change in the independent variable. The location of the interquartile range for each of these items was located higher in the scale range in the post program assessment compared to the pre-program assessment indicating that the program was beneficial to these outcomes. The odds (95% CI), p-value of moving up one level for self-esteem with completing the program was 4.89 (3.47, 6.88), p < 0.001, for self-efficacy was 4.65 (3.42, 6.32), p < 0.001, and for satisfaction with body image was 3.68 (2.66, 5.11), p < 0.001. Despite these promising results, the limitations of the pre-post intervention research design dictated that further work using randomised trial methodology was necessary to establish the efficacy of the “Girls on the Go!” program.

### Primary outcome measures

***The Rosenberg self-esteem scale*** (Rosenberg, 1965) was used to assess feelings of self-worth. This scale consists of ten items, each item is rated on a 4-point scale where 4 = “strongly agree”, 3 = “agree”, 2 = “disagree” and 1 = “strongly disagree”. An example of an item includes “I take a positive attitude towards myself”. Some items are negatively worded for instance “I certainly feel useless at times” and these have been reversed scored in final scoring process. Items were added up with possible scores for the scale ranged from 10 to 40 were higher scores are indicative of greater self-esteem. The Rosenberg self-esteem scale has been widely used and has been reported to have great internal consistency (Chronbach’s α = .88) and validity scores (Rosenberg, 1965; Rosenberg and Simmons, 1971; Simmons et al., 1973).

The ***eating disorders assessment*** [Clinical Interview Assessment, (CIA)] developed by Bohn and colleagues (Bohn et al., 2008) (16-items scale) was used to assess psycho-social impairment induced by eating disorders. Participants were asked to indicate over the past month, to what extent have their eating habits, exercising, or feelings about your eating, shape or weigh caused them impairment in three areas including cognitive “made you feel upset”, personal “Interfered with you doing things you used to enjoy” and social “made it difficult to eat out with others”. Each item is scored on a 4-point response format 0 = “not at all”, 1 = “slightly”, 2 = “moderately” and 3 = “a lot”. The scale scores ranged from 0 to 48, higher scores are indicative of greater impairment and eating disorders. This scale has demonstrated high internal consistency and test-retest reliability and good validity (Bohn et al., 2008).

### Secondary outcome measures

***Body satisfaction*** was measured by the body esteem scale developed by Katz-Mendelson and White (1982) which was found to be a reliable instrument with children as young as 7 years old. The 24 items in this scale encompass how a person values their appearance and body. There are equal numbers of positive and negative items and participants are asked to agree or disagree with each item. An example of a positive item is “I’m proud of my body” and a negative item is “I wish I was thinner”. The scale is scored by counting the number of responses indicating positive body esteem (maximum score is 24). A high score on the scale reflects higher body satisfaction.

***Body Dissatisfaction*** was measured with a subscale of the Eating Disorders Inventory (Garner et al., 1983). The scale contained 9 items designed to measure dissatisfaction with specific body parts. Examples of items include “I think my hips are too big” and “I think that my stomach is just the right size”. Responses were rated on a 6 point scale where 1 = never and 6 = always. Scores ranged from 0 to 54, with higher scores indicating higher dissatisfaction.

***Self-efficacy*** was measured by a self-efficacy scale designed to assess individuals’ sense of competence in particular areas. We used Froman and Owen’s (1991) health self-efficacy measure designed for use with high school students. The 43 item scale has two subscales, *physical health self-efficacy* and *mental health self-efficacy*. Participants are asked to indicate their degree of confidence where 1 = “a little” and 5 = “a lot” in their ability to complete a number of tasks. An example of task from *physical health self-efficacy scale* include “treating a fever” and *mental health self-efficacy scale* “maintaining a positive attitude towards school”. This scale was slightly refined to suit the current study’s participants. For example some items such as “avoiding illegal drugs”, were removed to make the scale appropriate for younger age participants. The final scale used had 37 items, scores for the scale ranged from 37 to 185 were higher scores are indicative of greater *physical health* or *mental health self-efficacy*.

***Dieting behavior*** was assessed by the Dutch Eating Behaviour Questionnaire for Children (van Strien and Oosterveld, 2008) comprised of three subscales including: *restrained eating*, *emotional eating* and *external eating*. The scale is designed to assess the degree of concern a participant has about dieting and restricting the amount of food they may otherwise eat. There are a total of 20 items. Participants are asked to circle either yes, no or sometimes to a statements such as *restrained eating subscale*: “If you have eaten too much do you eat less than usual the next day?”; *emotional eating subscale*, “if you feel depressed do you get a desire for food?” and *external eating subscale* “do you feel like eating whenever you see or smell food?”. Each item is scored on a 3-point response format no (0), sometimes (1), yes (2). The scores are then summed up and higher scores represent higher dieting behaviour. Scores range from 0–48. This scale has demonstrated excellent reliability, Cronbach’s α = 0.80 *restrained eating*, Cronbach’s α = 0.81 *emotional eating*, and Cronbach’s α = 0.74 *external eating*, (van Strien et al., 1986; van Strien and Oosterveld, 2008) and has been widely used (Higgins and Gray, 1998; Carter et al., 1997; Higgins and Altman, 2008).

### Procedure

#### Ethics and trial registration

The randomised control trial was prospectively registered with the Australian and New Zealand Control Trial Registry (ACTR 12610000513011). The trial was conducted in two phases that comprised Study 1 and Study 2.

Study 1 was prospectively registered with the Australian and New Zealand Control Trial Registry (ACTRN12610000513011). We then received notification of further funding to conduct this and Study 2. We submitted an amendment to our ethics application (ethics approval 10119B) to conduct Study 2 with the Monash Health Human Research Ethics Committee as the population of Study 2 and study design only varied slightly from Study 1 (Study 1 involved high school students and had a stepped wedge design of 3 schools being provided with the intervention per term, while Study 2 involved primary school students and had a stepped wedge design of 2 schools being provided with the intervention per term). We then amended the Australian and New Zealand Control Trial Registry so that the sample size listed on the registry was increased from 60 to 120 participants.

#### Study 1

High schools that contacted the Greater Dandenong Community Health Service in 2010 to receive the “Girls on the Go!” program that year were notified of the research and that their school’s placement on the waiting list that year would be determined at random. School welfare officers and teachers forwarded to the Greater Dandenong Community Health Service the list of student names who they determined met the study and program inclusion criteria. An investigator (LT) gained consent from participant’s parents (with assent from the participant) to participate in the research from all referred students. This investigator administered the data collection surveys at baseline and follow-up assessments at the time points indicated (Figures 1 and 2). The program was provided to schools in the order determined by the matched, random allocation sequence.

#### Study 2

The same recruitment and data collection approaches were used for study 2, though with recruitment derived from primary schools, and the addition of one further follow-up assessment.

### Analyses

The effect of being exposed to the “Girls on the Go!” program will be examined using a linear mixed model analysis approach, suitable for analysing longitudinal data where there is likelihood of missing data or loss to follow-up. The model that will be constructed to conduct this analysis will require construction of an independent variable, labelled “Girls on the Go”. This variable will be coded a 0 for observations where the group had not yet been exposed to the “Girls on the Go!” intervention (e.g. In study 1; at baseline for all schools and also at the first follow-up assessment but only for schools in the “Waiting List” period–see Figure 1), and 1 for observations where the participants had already received the “Girls on the Go!” intervention (e.g. In study 1; at the first follow-up assessment for schools in the first “Girls on the Go” period and also for all schools at the second follow-up assessment). Raw summative outcome measurement scores will be used as the dependent variables.

The mixed model analysis will involve investigating the effect of the “Girls on the Go” variable on the dependent variable, adjusting for assessment number (to account for the effect of time). The “Girls on the Go” variable will be entered into the model as a fixed factor, while assessment, student and school will be entered as random factors. Assessment will be nested within student which will be nested within school which will create a 3-level model that takes into account the longitudinal, clustered nature of data collected in this study. Maximum likelihood methods of estimation will be employed to address issues of missing data under the assumption of Missing At Random.

To investigate whether the effect of the “Girls on the Go!” program was sustained, a “Girls on the Go” by assessment interaction effect will be added to the same analysis models. A significant interaction effect in these models will indicate whether any changes brought about at the initial post-“Girls on the Go!” follow-up assessment were sustained. We will also examine the data from the group that received the “Girls on the Go!” program first and compare student results between the first and subsequent post-intervention follow-up assessments in isolation using linear regression clustering data by individual student.

### Power analysis

Analysis of data from the pilot study indicated that the effect of the “Girls on the Go!” program on participant self-esteem was very strong (ie. Change in outcome is 1.5 times the width of the standard deviation). Assuming 80% power, only 10 participants per group will be required in a standard randomised trial with randomisation of individual participants. This study will be a cluster randomised trial however to account for a variance inflation factor (design effect) of 3 (where average size per cluster is n =10, and the ICC is assumed to equal 0.33) then a total of 120 participants in both the primary (n = 60) and secondary (n = 60) schools will be required.

## Discussion

Low self-esteem and its associate health consequences such as body dissatisfaction and eating disorders are important societal, cultural, familial and individual matters. They demand urgent attention, greater resources and evidenced based approaches to both assist young people, and girls in particular with low self-esteem and body image concerns and to prevent others from its development. High self-esteem is known to be a protective factor against the development of body dissatisfaction, and disordered eating and therefore this project may have the potential to contribute to the field of prevention of disordered eating. “Girls on the Go!” was developed to deal with these particular issues. To our knowledge this is the first group-based, professional-led, targeted program conducted outside the school environment amongst school age young people with such culturally diverse backgrounds to be evaluated.

This research will enable clinicians to evaluate a new community preventative intervention and thus to apply research into clinical practice. Before the expansion of “Girls on the Go!” it should be subjected to evaluation to demonstrate that it does achieve its objectives. These findings will indicate if the “Girls on the Go!” program may be successfully used and applied in a culturally diverse community and with disadvantaged participants. The results of the two individual studies will allow for comparison of the intervention’s effect at various age groups (i.e. primary school vs secondary schools age young women). Furthermore, “Girls on the Go!” targets girls who have diverse body shapes and sizes and this control trial results will indicate if a holistic approach focused on self-esteem will lead to overall improved health and wellbeing. Therefore, these findings will support a relatively new paradigm shift where self-esteem enhancement and overall health and well-being are emphasized and used as a prevention strategy and it will potentially support the notion that improving self-esteem would lead to improved self-efficacy and body satisfaction in young women (O’Dea and Maloney, 2000; O’Dea, 2005, 2000b; Bacon and Aphramor, 2011).

Information collected by the present research, that documents the development of a preventative intervention originated from a health service delivery, and evaluation of these data, will contribute new information to the field of prevention of eating disorders and health promotion delivery.

## Authors’ original submitted files for images

Below are the links to the authors’ original submitted files for images. Authors’ original file for figure 1 Authors’ original file for figure 2
