# Supplementary material for: Investigation of the effectiveness of the “Girls on the Go!” program for building self-esteem in young women: trial protocol
Source: Springerplus. 2013 Dec 19;2(1):683. doi: 10.1186/2193-1801-2-683 (PMC3877412; doi:10.1186/2193-1801-2-683)
Supplement: Supplementary file 2 — Authors’ original file for figure 2 [file 40064_2013_751_MOESM2_ESM.pdf]

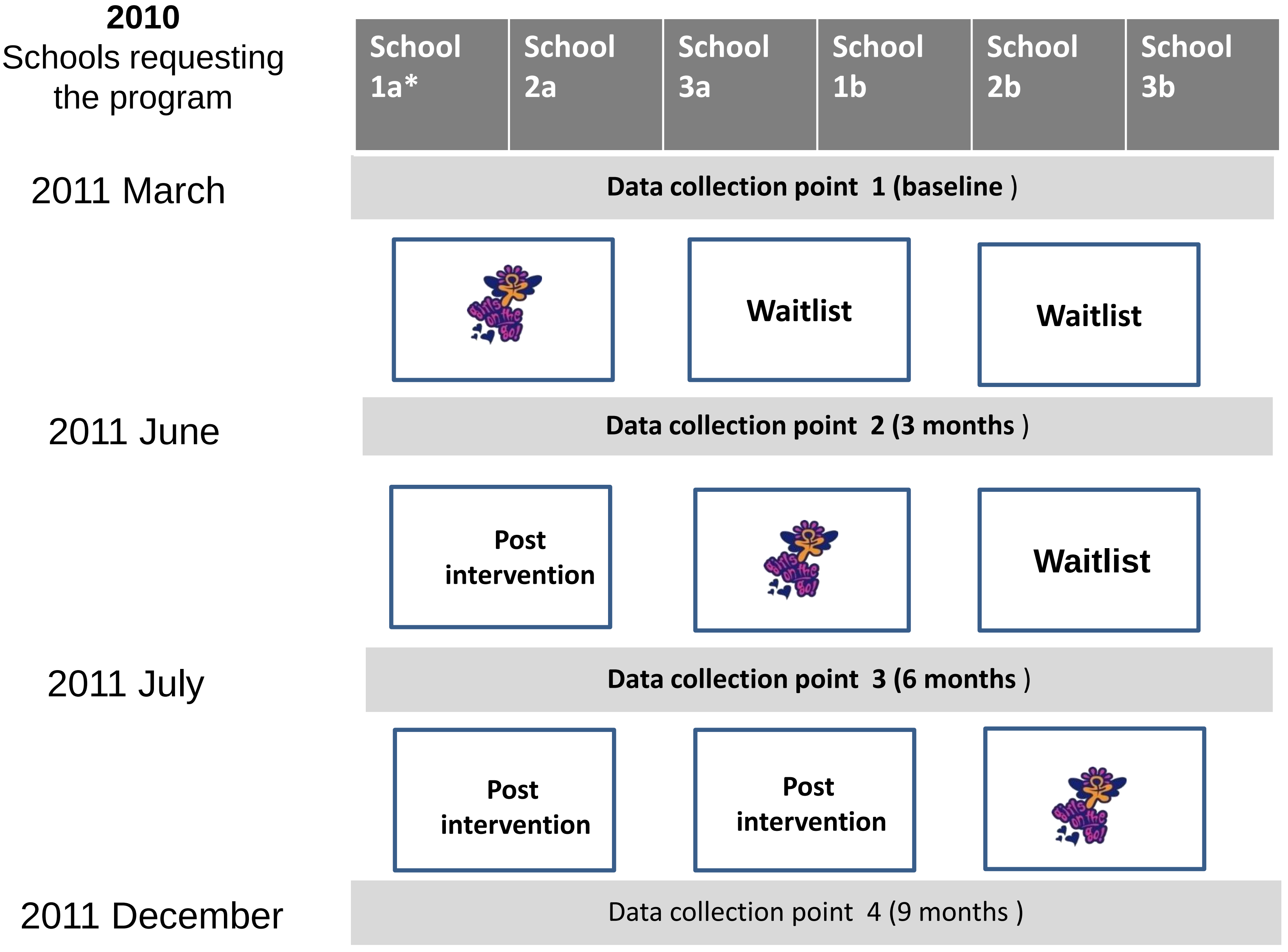
2010
Schools requesting the program
School 1a*
School 2a
School 3a
School 1b
School 2b
School 3b
2011 March
Data collection point 1 (baseline)
Waitlist
Waitlist
2011 June
Data collection point 2 (3 months)
Post intervention
Waitlist
2011 July
Data collection point 3 (6 months)
Post intervention
Post intervention
2011 December
Data collection point 4 (9 months)
